# Mechanisms of Reactive Oxygen Species Generated by Inorganic Nanomaterials for Cancer Therapeutics

**DOI:** 10.3389/fchem.2021.630969

**Published:** 2021-03-18

**Authors:** Lizhen Zhang, Chengyuan Zhu, Rongtao Huang, Yanwen Ding, Changping Ruan, Xing-Can Shen

**Affiliations:** ^1^ State Key Laboratory for Chemistry and Molecular Engineering of Medicinal Resources, School of Chemistry and Pharmaceutical Science, Guangxi Normal University, Guilin, China; ^2^ Guilin Normal College, Guilin, China

**Keywords:** reactive oxygen species, cancer therapeutics, inorganic nanomaterials, generation mechanisms, photodynamic therapy, chemodynamic therapy

## Abstract

Recently, inorganic nanomaterials have received considerable attention for use in biomedical applications owing to their unique physicochemical properties based on their shapes, sizes, and surface characteristics. Photodynamic therapy (PDT), sonodynamic therapy (SDT), and chemical dynamic therapy (CDT), which are cancer therapeutics mediated by reactive oxygen species (ROS), have the potential to significantly enhance the therapeutic precision and efficacy for cancer. To facilitate cancer therapeutics, numerous inorganic nanomaterials have been developed to generate ROS. This mini review provides an overview of the generation mechanisms of ROS by representative inorganic nanomaterials for cancer therapeutics, including the structures of engineered inorganic nanomaterials, ROS production conditions, ROS types, and the applications of the inorganic nanomaterials in cancer PDT, SDT, and CDT.

## Introduction

Reactive oxygen species (ROS) are reactive radicals or non-radical oxygen-containing molecules with reactive properties. ROS primarily include singlet oxygen (^1^O_2_), hydrogen peroxide (H_2_O_2_), hydroxyl radical (•OH), and superoxide anion (O_2_^•−^) (Yang et al., 2019). Intrinsic ROS are derived from various metabolic reactions that mainly occur in the mitochondria. These reactions are essential for the development of living organisms. The overexpression of ROS causes oxidative stress and can lead to the destruction of cellular components. This property is beneficial for the design of ROS-related inorganic nanomaterials for therapeutic applications, including PDT, SDT, and CDT (Yang et al., 2019). PDT is a therapeutic strategy in which a photosensitizer (PS) or a photosensitizing nanoplatform selectively absorbs light of a specific wavelength to produce ROS, which can cause cell death (Liu et al., 2020a). SDT employs high-penetrating acoustic waves to trigger the ultrasound-responsive materials generating ROS for the cancer treatment (Wu et al., 2019). CDT agents can effectively kill cancer cells by catalyzing H_2_O_2_ production in the tumor microenvironment (TME) (Lin et al., 2018). Therefore, ROS-related cancer therapeutics show potential to significantly enhance the precision and efficacy of cancer therapeutics.

Given the tremendous progress in the field of nanotechnology, a large number of inorganic nanomaterials with intrinsic ROS-regulating properties have been prepared for widespread biomedical applications. This mini review provides an overview of the mechanisms by which representative inorganic nanomaterials generate ROS for cancer therapeutics, including the structures of engineered inorganic nanomaterials, ROS production conditions, ROS types, and the applications of the nanomaterials in cancer PDT, SDT, and CDT.

## Inorganic Nanomaterials and Their Mechanisms of ROS Generation

Remarkable progress has been made with respect to inorganic nanomaterials that can facilitate intrinsic ROS generation. However, the mechanisms of ROS generation by representative inorganic nanomaterials are rarely summarized. This section discusses the mechanisms of ROS generation by inorganic nanomaterials including carbon nanomaterials, semiconductor nanomaterials, defective inorganic nanomaterials, and Fenton agents.

### Carbon Nanomaterials Generate ROS Mechanisms

Carbon nanomaterials are a class of carbonaceous materials, including fullerenes, carbon dots (CDs), carbon nanotubes, carbon nanohorns, graphene (GR), and graphene oxide (GO) (Panwar et al., 2019). The fundamental structures of carbon nanomaterials are based on sp^2^-hybridized carbon atoms that are generally arranged in hexagonal lattices which can form various topological distortions (Karousis et al., 2016). This endows carbon nanomaterials with unique physical and chemical properties (Panwar et al., 2019), leading to numerous applications in biosensing, bioimaging, and biomedicine (Cui et al., 2018). Since many carbon nanomaterials have shown great potential for ROS generation, they are expected to be applicable as activatable PSs for PDT. However, the mechanisms of ROS generation by different carbon materials are diverse.

**FIGURE 1:**
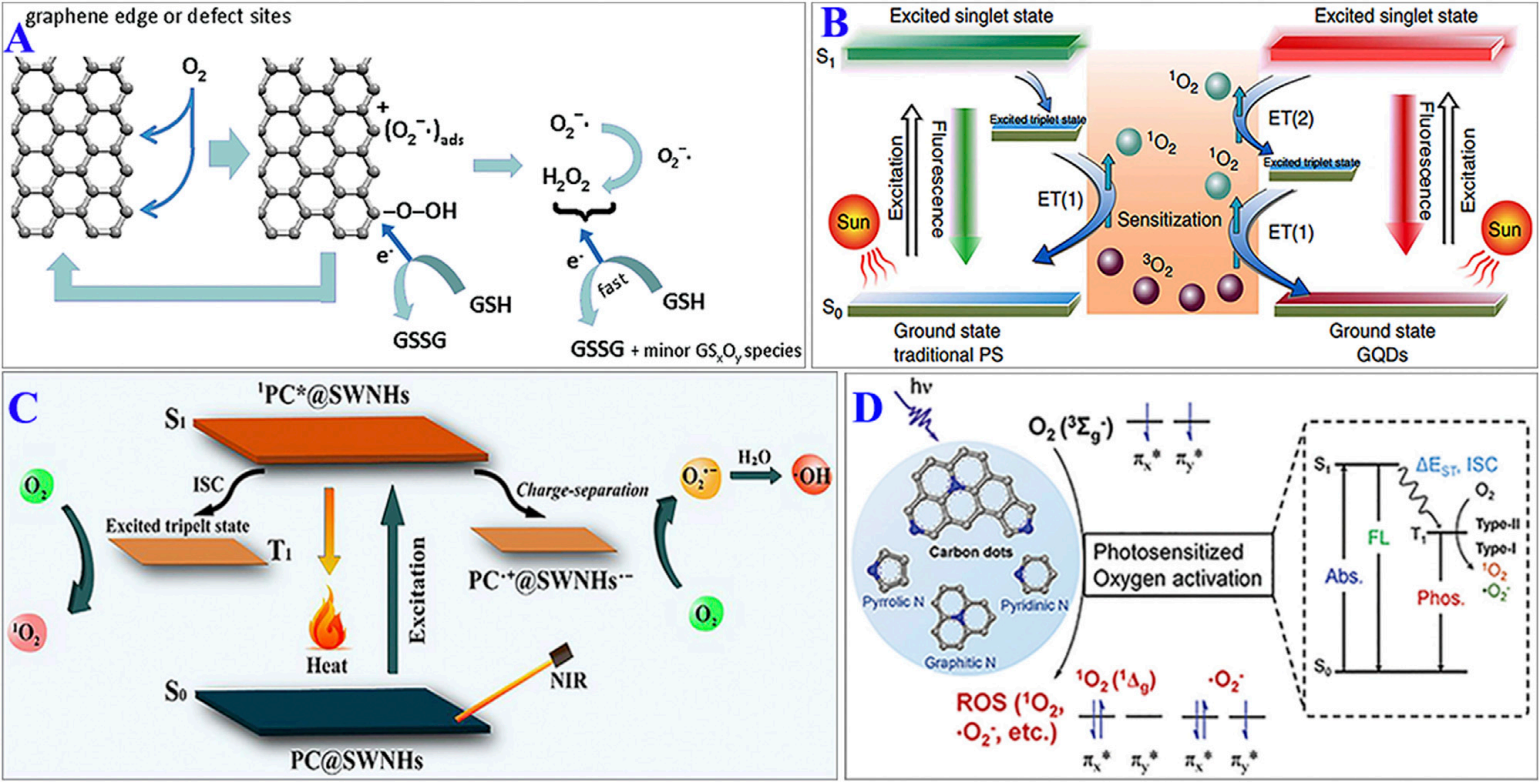
**(A)** Defect sites in carbon nanomaterials and ROS generation. Reprinted with permission from Liu et al. Copyright (2011) Wiley-VCH. **(B)** Schematic of the mechanisms of ^1^O_2_ production by traditional PDT agents (left) and GQDs (right). Reprinted with permission from Ge et al. Copyright (2014) Nature Publishing Group. **(C)** Schematic of the mechanism of ROS generation by PC@SWNHs under 650-nm laser irradiation. Reprinted with permission from Lin et al. Copyright (2019) Elsevier. **(D)** Nitrogen-doped CDs for photosensitized oxygen activation. Reprinted with permission from Wu et al. Copyright (2020) The Royal Society of Chemistry.

Liu et al. investigated the relationship between oxidative stress and the surface areas of various graphenic carbon materials. They found that the reaction between graphenic carbon materials and dissolved dioxygen usually takes place at the graphenic edge or defect sites, generating ROS such as O_2_^•−^ and H_2_O_2_ *via* a heterogeneous process; these ROS then oxidize GSH (Figure 1A) (Liu et al., 2011). Dong et al. also discovered the electrocatalytic activity depended on the content of topological C defects (Dong et al., 2020). When two-dimensional graphene sheets were converted to zero-dimensional CDs, phenomena associated with edge effects and quantum confinement were observed due to size effects. In 2014, Ge et al. developed a type of graphene quantum dot (GQD) with PDT properties. The GQDs demonstrated an ^1^O_2_ quantum yield of more than 1.3 *via* a multistate sensitization process. As shown in Figure 1B, the high ^1^O_2_ yield of the GQDs is derived from two pathways: the conventional pathway, namely, energy transfer from the excited triplet state (T_1_) to the ground state (S_0_); and energy transfer from the excited singlet state (S_1_) to T_1_ *via* the intersystem crossing transition. Consequently, the ^1^O_2_ quantum yield reached 1.3 (Ge et al., 2014). Subsequently, Shen’s group prepared hyaluronic acid (HA)-derived CDs which can produce O_2_^•−^ *via* a photoinduced electron transfer process (Zhang et al., 2018). These studies demonstrate that pure CDs can produce ROS upon photoexcitation owning to the band-edge defect effect. Therefore, it can improve the ROS generation by adjusting the edge defects of carbon materials.

Surface functionalization is an effective strategy to improve the photosensitization performance of CDs. For example, Shen et al. used tetrasulfonic acid copper phthalocyanine (TSCuPc) as a PS and GR or single-walled carbon nanohorns (SWNHs) as PS carriers to prepare water-soluble GR-TSCuPc and SWNHs-TSCuPc nanohybrids. These GR-TSCuPc and SWNHs-TSCuPc nanohybrids demonstrated excellent ROS production ability. When TSCuPc is photoexcited, electron transfer occurs from the excited state of TSCuPc to the SWNHs (or GR). This results in the formation of a charge-separated state (SWNHs^•−^–TSCuPc^•+^ or GR^•−^–TSCuPc^•+^), which further transfers the electrons to surrounding O_2_ to generate O_2_^•−^ and then •OH (Jiang et al., 2014a; Jiang et al., 2014b). In another works, the functionalized SWNHs exhibited the similar •OH generation process. Meanwhile, ^1^O_2_ was generated *via* the conventional energy transfer pathway from T_1_ to S_0_ (Figure 1C) (Lin et al., 2019a). These works indicated surface functionalization can induce ROS generation.

Heteroatom doping is an effective approach to improve the surface functionalization of CDs, which is important for ROS generation. Wu’s group synthesized a series of N-doped CDs (N-CDs) with a variety of N types to explore how the speciation of the nitrogen dopant affected the oxygen photosensitization performance of the N-CDs. It is found that among N types, graphitic N and pyrrolic N had the greatest effects on the photosensitization properties of CDs. The presence of graphitic N in N-CDs can reduce the energy gap between S_1_ and T_1_ (Δ*E*_ST_) to promote ISC, which can improve the photosensitization performance. Pyrrolic N was the main site of oxygen adsorption, which contributed to the highly efficient oxygen photosensitization of N-CDs by shortening the distance between oxygen and the N-CDs (Figure 1D) (Wu et al., 2020). The Cu-doped CDs can also effectively generate ROS (Wu et al., 2015; Guo et al., 2018). These results indicate that heteroatom doping is an effective strategy to improve ROS generation by CDs.

### Semiconductor Nanomaterials Generate ROS Mechanism

**FIGURE 2:**
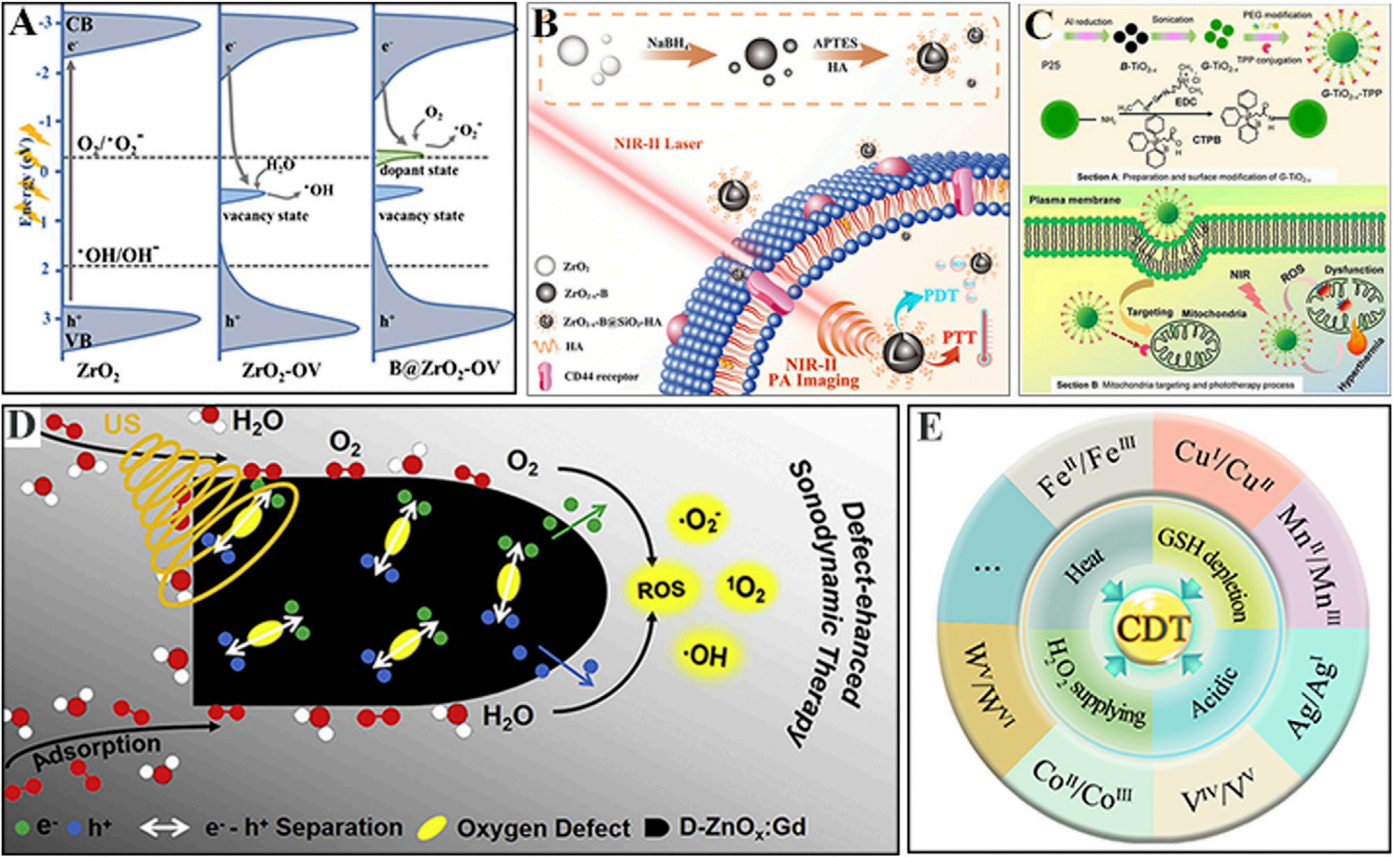
**(A)** Band edge alignment of B@ZrO_2_−OV for visible-to-NIR photon harvesting. Reprinted with permission from Qin et al. Copyright (2020) American Chemical Society. **(B)** Design of NIR-responsive ZrO_2-x_−B@SiO_2_−HA for cancer phototherapy. Reprinted with permission from Zhu et al. Copyright (2020) The Royal Society of Chemistry. **(C)** Design and schematic of G-TiO_2-x_-TPP with excellent NIR absorption for cancer treatment. Reprinted with permission from Mou et al. Copyright (2017) Ivyspring. **(D)** The proposed mechanism of defect-enhanced sonodynamic ROS generation in D−ZnOx:Gd. Reprinted with permission from Liu et al. Copyright (2020b) Elsevier. **(E)** Summary of metal-based inorganic nanomaterials for CDT and strategies for improving CDT.

Semiconductor nanomaterials have excellent prospects for applications in photocatalysis due to their distinct physical and chemical properties. It is found that semiconductors can absorb sunlight to catalyze different photochemical reactions, and ROS are the primary intermediates in photocatalytic reactions (Nosaka and Nosaka, 2017). The generated ROS in photocatalytic reaction mechanism are executed by redox reactions *via* oxygen and water with photogenerated electrons (e^−^) and holes (h^+^) on the surface of the semiconductor photocatalyst. Thus, semiconductor photocatalysts have great potential for use as PSs for PDT. Studies have extended the optical responses of semiconductor photocatalysts into the visible and even NIR regions by introducing doping atoms or surface defects to modify the electronic band structure and improve the overall catalytic efficiency. For example, Qin et al. prepared a novel photocatalyst B@ZrO_2_−OV that can achieve photon harvesting in the full spectrum from visible light to NIR light (Figure 2A). The doped B atoms and OVs into ZrO_2_ result in more impurity electronic states and narrow bandgap, resulting in full spectrum absorption, and can efficiently produce electron−hole pairs under the NIR light irradiation, and react with adsorbed O_2_/H_2_O on the surface to produce O_2_^•−^ and •OH (Qin et al., 2020). The surface modification or functionalization of semiconductors can also improve their biocompatibility and delivery efficiency in tumors. This full-spectrum responsive B@ZrO_2_−OV was modified with HA to obtain ZrO_2−x_−B@SiO_2_−HA, which exhibits improved water dispersibility and the ability to target tumor cells (Figure 2B). The obtained ZrO_2−x_−B@SiO_2_−HA also shows promise for high-resolution photoacoustic imaging along with remarkable synergistic PTT/PDT treatment effect upon 1064-nm laser irradiation (Zhu et al., 2020). Mou et al. developed green titania (G−TiO_2−x_) with excellent NIR absorption by altering the lattice of black titania (B−TiO_2−x_) *via* strong ultrasonication, and further conjugated G−TiO_2−x_ with triphenylphosphonium (TPP) ligand to obtain G−TiO_2−x_−TPP, which exhibits mitochondria-targeting ability for combined PTT/PDT precise cancer therapy (Figure 2C) (Mou et al., 2017). These works have significantly broadened the application of semiconductors in nanomedicine through reasonable design and control of the nanostructures and physicochemical properties of semiconductor-based nanomaterials.

### Defective Inorganic Nanomaterials for ROS-Generation

In addition to carbon nanomaterials and semiconductors, defective inorganic nanomaterials have also been shown to generate ROS for cancer therapeutics. In the defect engineering of inorganic nanomaterials, the particle size, morphology, and crystal defects are controlled with appropriate synthetic methods so that the ROS generation activity can be tuned (Qin et al., 2020). This strategy has been used to produce many defective inorganic nanomaterials for ROS-related cancer therapeutics (Han et al., 2018). Therefore, the mechanism of defect-mediated tunable ROS generation by inorganic nanomaterials should be studied. Recently, Shen’s group successfully developed one type of brand-new artificial metalloprotein nanoanalogues (RuO_2_@OVA NAs) for enhanced photoimmunotherapy *via* the reasonable integration of defective RuO_2_ NPs and the model antigen ovalbumin (OVA) (Huang et al., 2020). The following possible mechanism of photoinduced ^1^O_2_ production by defective RuO_2_ NPs in RuO_2_@OVA NAs was proposed: O_2_ is sensitized to ^1^O_2_ by defects *via* energy transfer under 808-nm laser irradiation. This work significantly broadens the nanomedical applications of RuO_2_-based nanomaterials *via* the rational design of structural defects.

Defective inorganic nanomaterials are also employed as sonosensitizers to generate ROS for the cancer treatment through different mechanisms such as the sonoluminescence process, pyrolysis, and acoustic cavitation effects (Wu et al., 2019). Zhang et al. reported a novel defect-rich gadolinium (Gd)-doped zinc oxide nanobullet (D−ZnOx:Gd) sonosensitizer for cancer therapeutics (Liu et al., 2020b). The abundant oxygen defects promote the separation of electrons (e^−^) and holes (h^+^) from the energy band structure of D−ZnOx:Gd upon external ultrasound (US) irradiation, causing O_2_ to be more sensitized to electrons (Figure 2D). This endow D−ZnOx:Gd with high sonodynamic ROS generation ability for SDT. This work not only provides a new application for defect engineering in cancer therapeutics but also reveals the mechanism by which the oxygen defects enhance the efficiency of ROS generation. Furthermore, defective inorganic radiosensitizers operate by nonexclusive mechanisms to enhance radiation effects on cancer cells. Zhao group reported defect-abundant BiO_2−x_ nanosheets (NSs) as radiosensitizers with catalase-like activity for hypoxia alleviation and tumor radiotherapy (Liu et al., 2020c). The oxygen defects in the BiO_2−x_ NSs function as traps for electrons, which are easily transferred to O_2_ to form O_2_^•−^. This work provides a new strategy for ROS-related cancer therapeutics. The above studies clearly demonstrate that the defects in inorganic nanomaterials are critical for tunable ROS generation in cancer therapeutics. The mechanism of defect-mediated tunable ROS generation by inorganic nanomaterials can be summarized as follows. On the one hand, the defects introduce defect levels between the conduction band (CB) and the valence band (VB) of inorganic metal oxides. These defect levels reduce the bandgap and increase the edge of the VB. On the other hand, the existence of defects can result in easier to adsorb oxygen molecules or promote the holes and separation of electrons. Briefly, the presence of defects in inorganic nanomaterials allows O_2_ to be more easily sensitized to electrons, facilitating tunable ROS generation.

### Fenton Agents for ROS Generation

Fenton and Fenton-like reactions have attracted extensive attention as an emerging cancer treatment that can transfer endogenous hydrogen peroxide (H_2_O_2_) into highly cytotoxic •OH. As shown in Figure 2E, although the majority of existing Fenton agents for cancer therapy are iron-based inorganic nanomaterials, other inorganic redox-active transition metals such as Cu, Mn, Ag, V, Co, and W along with nanozymes (e.g., Fe_3_O_4_) have been used to induce Fenton-like reactions (Han et al., 2020). For example, Lin et al. showed that manganese dioxide (MnO_2_) has dual Fenton-like Mn^2+^ delivery and GSH depletion properties, and can thus be used as a smart Fenton-like agent to deliver •OH into cells to destroy the cellular antioxidant defense system and thereby enhancing the CDT effect (Lin et al., 2018). Bu et al. developed AFeNPs@CAI nanocomposites from amorphous iron nanoparticles (AFeNPs) loaded with carbonic anhydrase IX inhibitor (CAI). The release of CAI led to the accumulation of H^+^, which accelerated the Fenton reaction and amplified the oxidative damage of cells (Chen et al., 2019). Fenton-like reactions catalyzed by Cu^+^ are kinetically more favorable than those catalyzed by Fe^2+^ under neutral and weakly acidic conditions. To further improve the reaction efficiency of Cu**-**based nanomaterials, intracellular H_2_O_2_ may be challenging. Li et al. reported self-assembled copper amino acid mercaptide nanoparticles induce •OH generation *via* Fenton-like reaction through their sequential oxidation–reduction reactions with intracellular GSH “AND” H_2_O_2_ (Ma et al., 2019). Chen et al. reported the fabrication of copper peroxide (CP) nanodots that enhance CDT by self-supplying H_2_O_2_. In the TME, the acidic environment of endo/lysosomes accelerates the dissociation of CP nanodots, simultaneously releasing Cu^2+^ and H_2_O_2_, which improves the efficiency of the Fenton-like reaction (Lin et al., 2019b).

Nanozymes are a class of nanomaterials with inherently enzyme-like properties. In 2007, Yan et al. first reported that Fe_3_O_4_ nanoparticles have intrinsic peroxidase-mimicking activity (Gao et al., 2007). The Fenton reaction properties of Fe_3_O_4_ have been reported in cancer treatment. For example, Shi et al. simultaneously loaded the natural enzyme glucose oxidase (GOD) and nanozyme Fe_3_O_4_ into large pore-sized and biodegradable dendritic silica nanoparticles to construct sequential GFD nanocatalysts. GOD can effectively consume glucose in tumor cells while also producing a large amount of H_2_O_2_ and reducing the acidity, which are used in the Fenton-like reaction of the nanoenzyme Fe_3_O_4_ to generate •OH (Huo et al., 2017). In 2020, Chen et al. synthesized vanadium oxide–based nanozymes (TA@VOx NSs) could catalyze H_2_O_2_ to generate •OH. Importantly, the authors also demonstrated that heat can effectively improve the •OH generation efficiency of Fenton-like reaction (Chen et al., 2020). The above studies show that various inorganic nanomaterials and strategies have been applied to enhance the efficiency of Fenton and Fenton-like reactions for ROS generation. These efforts can greatly promote the clinical application of Fenton and Fenton-like reactions in cancer treatment.

## Conclusion and Challenges

This brief overview focuses on the mechanisms of ROS generation by representative inorganic nanomaterials for cancer therapeutics. Although various inorganic nanomaterials have been reported to generate ROS, several remaining challenges limit the precision and efficacy of ROS-based cancer therapeutics:

1. Inadequate accumulation in cancer tissues and relevant systemic toxicity. Although inorganic nanomaterials can effectively catalyze the production of ROS, their biodegradation mechanism *in vivo* remains unclear, and the lack of specificity in the delivery and distribution of inorganic nanomaterials in normal tissues is still a problem that needs to be solved (Qian et al., 2019).
2. The TME allows CDT, PDT, and SDT to be selective and minimally invasive; however, the hypoxic conditions, limited concentration of intracellular H_2_O_2_, and overexpression of GSH in the TME significantly reduce the efficacy of ROS-based cancer therapy. Therefore, it is necessary to develop an intelligent strategy to modulate the TME (Yin et al., 2019).
3. The ROS is a double-edged sword. On the one hand, ROS can induce cell apoptosis in PDT, SDT, and CDT. On the other hand, the ROS produced by foodborne CDs could disrupt the mitochondrial membrane and changed the transcriptional level of genes related to ROS, which will be the potential health risk after digestion and absorption (Wang et al., 2020).

Moreover, organic nanomaterials have been reported to generate ROS which were mainly through fluorescence resonance energy transfer (FRET)-mediated direct or indirect sensitization of oxygen molecules process (Ng and Zheng, 2015). Therefore, a comprehensive understanding of the mechanism of ROS generation will be conducive to the design and construction of nanomaterials that will play important roles in precise and effective cancer therapeutics.
